# Supplementary material for: Stability of Leaf Yerba Mate (Ilex paraguariensis) Metabolite Concentrations over the Time from the Prism of Secondary Sexual Dimorphism
Source: Plants (Basel). 2023 Jun 2;12(11):2199. doi: 10.3390/plants12112199 (PMC10255638; doi:10.3390/plants12112199)
Supplement: Supplementary file 1 [file plants-12-02199-s001.zip › plants-2420356-supplementary.pdf]

Supplementary Information for Original Paper:

Stability of Leaf Yerba Mate (*Ilex paraguariensis*) Metabolite Concentrations over the Time from the Prism of Secondary Sexual Dimorphism

Miroslava Rakocevic [1,2,*], Aline de Holanda Nunes Maia [2], Marcus Vinicius de Liz [3], Rafaela Imoski [3], Cristiane Vieira Helm [1], Euclides Lara Cardozo Junior [4] and Ivar Wendling [1]

[1] The Laboratory of Plant Breeding, Embrapa Florestas, Colombo 83411-000, PR, Brazil
[2] Statistical Research Group, Embrapa Meio Ambiente, Jaguariúna 13918-110, SP, Brazil
[3] Research Group on Water and Wastewater Advanced Treatment Technologies, Department of Chemistry and Biology, Federal University of Technology-Paraná, Curitiba 81280-340, PR, Brazil
[4] Department of Pharmacy, UNIPAR, Paranaense University, Toledo 85903-170, PR, Brazil
* Correspondence: mima.rakocevic61@gmail.com; Tel.: +55-19-97161-8918

Table S1. The influence of progeny, gender, and their interaction on the contents of theobromine, caffeine, chlorogenic and caffeic acids (%, w/w) in leaves of yerba-mate plants, as measured by ANOVA´s F-test *p*-values. Plants were originated from four provenances, with leaf collections performed in three phenophases, at 12, 18 and 24 months after the pruning, corresponding to the first winter (WinP-12), summer (SumP-18), and second winter growth pause (WinP-24).

			Nominal significance levels (*p*-values)			
Phenophase	Provenance	Source	Theobromine	Caffeine	Chlorogenic acid	Caffeic acid
WinP-12	Ivaí	Progeny (P)	0.4229 ns	**0.0008** ***	**0.0410** *	0.2489 ns
		Gender (G)	0.1277 ns	0.8410 ns	0.8088 ns	0.6028 ns
		P x G	0.7715 ns	0.4513 ns	0.1181 ns	0.6827 ns
	Barão de Cotegipe	Progeny (P)	**0.0002**	**0.0016** **	0.8354 ns	0.5377
		Gender (G)	0.6830	0.1506 ns	**0.0604** ·	0.7353
		P x G	**0.0005** ***	0.1594 ns	0.5343 ns	**0.0447** *
	Quedas do Iguaçu	Progeny (P)	**0.0191**	**0.0586**	0.8988 ns	0.3511
		Gender (G)	0.3394	0.4469	0.4673 ns	0.2149
		P x G	**0.0234** *	**0.0233** *	0.3728 ns	**0.0337** *
	Cascavel	Progeny (P)	0.6935 ns	**0.0855** ·	0.7041 ns	0.5122 ns
		Gender (G)	0.6717 ns	0.5653 ns	0.6415 ns	0.8112 ns
		P x G	0.9013 ns	0.7763 ns	0.1009 ns	0.3718 ns
SumP-18	Ivaí	Progeny (P)	0.3960 ns	**0.0109** *	0.3219 ns	0.2729 ns
		Gender (G)	0.4457 ns	0.6466 ns	0.8182 ns	0.2765 ns
		P x G	0.3327 ns	0.3264 ns	0.4143 ns	0.2077 ns
	Barão de Cotegipe	Progeny (P)	0.4583 ns	0.1077 ns	**0.0773** ·	**0.0011** **
		Gender (G)	0.7044 ns	0.2355 ns	0.3830 ns	0.5286 ns
		P x G	0.3423 ns	0.5414 ns	0.2763 ns	0.1154 ns
	Quedas do Iguaçu	Progeny (P)	0.6916 ns	0.7744 ns	0.1618 ns	**0.0591** ·
		Gender (G)	0.7152 ns	0.9508 ns	0.3919 ns	**0.0096** **
		P x G	0.5999 ns	0.4730 ns	0.6308 ns	0.6371 ns
	Cascavel	Progeny (P)	**0.0087** **	0.6089 ns	**0.0181** *	0.5969 ns
		Gender (G)	0.7088 ns	0.8189 ns	0.1184 ns	0.1354 ns
		P x G	0.4693 ns	0.5739 ns	0.4907 ns	0.4576 ns
WinP-24	Ivaí	Progeny (P)	**0.0030** **	**0.0001**	**0.0899** ·	0.1281 ns
		Gender (G)	0.5108 ns	0.2675	0.6413 ns	0.6291 ns
		P x G	0.8637 ns	**0.0786** ·	0.5765 ns	0.9704 ns
	Barão de Cotegipe	Progeny (P)	**0.0906** ·	**0.0564** ·	**0.0147**	0.1712 ns
		Gender (G)	0.4836 ns	0.2246 ns	**0.0208**	0.4359 ns
		P x G	0.8132 ns	0.2869 ns	**0.0940** ·	0.9985 ns
	Quedas do Iguaçu	Progeny (P)	**0.0599** ·	0.4052 ns	0.3118 ns	0.7744 ns
		Gender (G)	0.8050 ns	0.2494 ns	0.5451 ns	0.8850 ns
		P x G	0.5277 ns	0.3206 ns	0.2995 ns	0.9229 ns
	Cascavel	Progeny (P)	**0.0318**	0.2233 ns	0.3008 ns	0.9269 ns
		Gender (G)	0.2003	0.4407 ns	0.9638 ns	0.7437 ns
		P x G	**0.0610** ·	0.8191 ns	0.2881 ns	0.8538 ns

Significance levels ('***'<0.001, '**'<0.01, '*'<0.05, '·' <0.10) are also indicated. F-test *p*-values corresponding to ANOVA´s factors significant at 0.10 were highlighted in bold. Warning: whenever the P x G interaction was significant, the *p*-values associated with the principal factors (progeny or gender) were meaningless.
